# Supplementary material for: Complex‐centric proteome profiling by SEC‐SWATH‐MS
Source: Mol Syst Biol. 2019 Jan 14;15(1):e8438. doi: 10.15252/msb.20188438 (PMC6346213; doi:10.15252/msb.20188438)
Supplement: Supplementary file 8 — Dataset EV7 [file MSB-15-e8438-s008.zip › feature_plots_string/Q9H008.pdf]

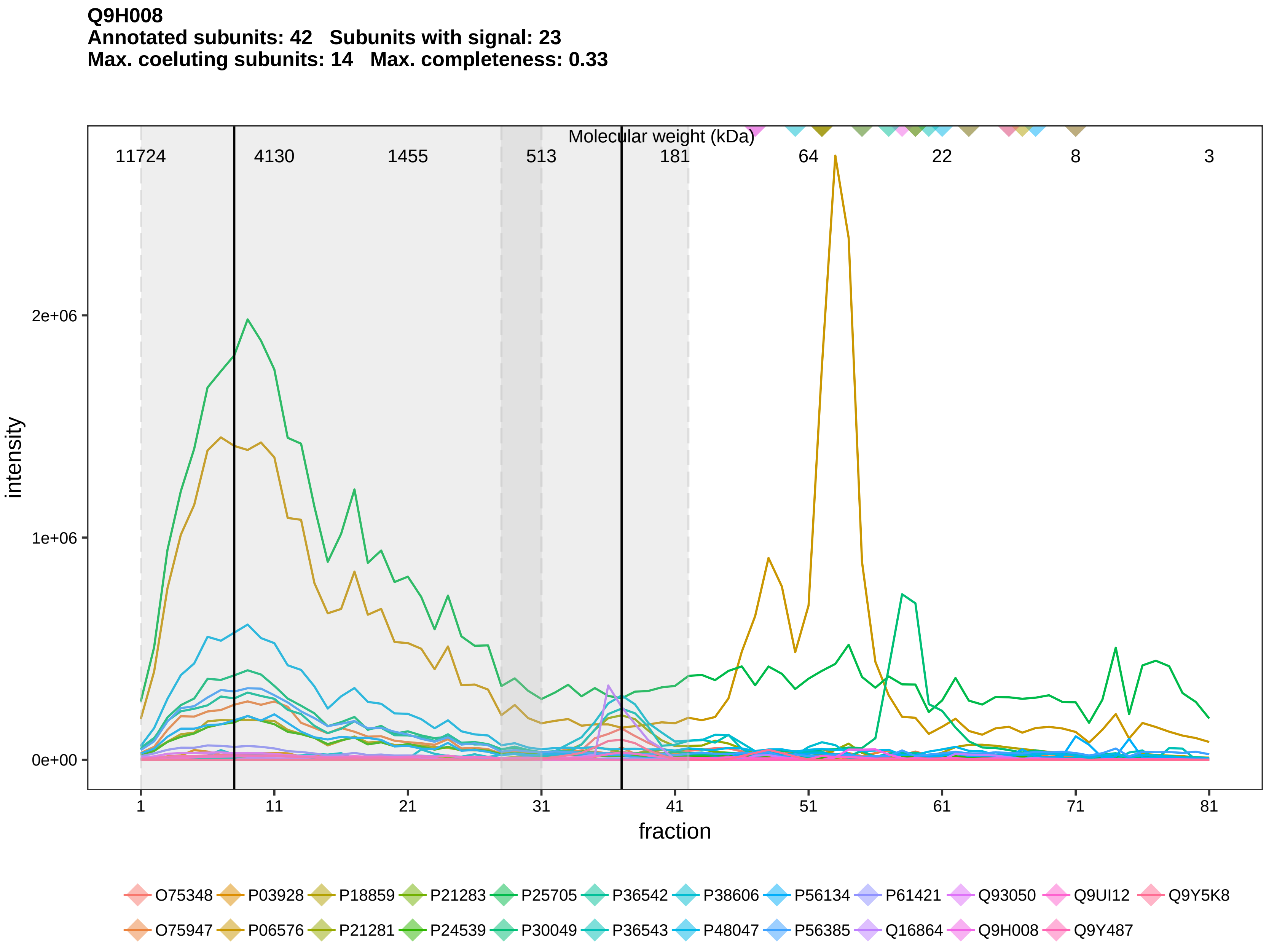
Q9H008
Annotated subunits: 42   Subunits with signal: 23
Max. coeluting subunits: 14   Max. completeness: 0.33
Molecular weight (kDa)
11724
4130
1455
513
181
64
22
8
3
intensity
2e+06
1e+06
0e+00
1
11
21
31
41
51
61
71
81
fraction
O75348
P03928
P18859
P21283
P25705
P36542
P38606
P56134
P61421
Q93050
Q9UI12
Q9Y5K8
O75947
P06576
P21281
P24539
P30049
P36543
P48047
P56385
Q16864
Q9H008
Q9Y487
